# Serum and peritoneal fluid concentrations of soluble human leukocyte antigen, tumor necrosis factor alpha and interleukin 10 in patients with selected ovarian pathologies

**DOI:** 10.1186/s13048-017-0320-9

**Published:** 2017-04-04

**Authors:** Olimpia Sipak-Szmigiel, Piotr Włodarski, Elżbieta Ronin-Walknowska, Andrzej Niedzielski, Beata Karakiewicz, Sylwia Słuczanowska-Głąbowska, Maria Laszczyńska, Witold Malinowski

**Affiliations:** 1 Department of Obstetrical and Gynecological Nursing, Pomeranian Medical University, 48 Żołnierska, 71-210 Szczecin, Poland; 2 Clinical Hospital SPS ZOZ “Zdroje”, Mączna 4, 70-780 Szczecin, Poland; 3 Clinic of Maternofetal Medicine and Gynecology, Pomeranian Medical University in Szczecin, Unii Lubelskiej 1, 71-242 Szczecin, Poland; 4 Public Health Department, Pomeranian Medical University in Szczecin, Żołnierska 48, 71-210 Szczecin, Poland; 5 Deparment of Physiology, Pomeranian Medical University in Szczecin, al. Powstańców Wlkp. 72, 70-111 Szczecin, Poland; 6 Department of Histology and Developmental Biology, Pomeranian Medical University in Szczecin, Żołnierska 48, 71-210 Szczecin, Poland

**Keywords:** Ovarian cancer, Endometriosis, Ovarian cysts, IL-10, TNF-alpha, sHLA-G, Tumor escape

## Abstract

**Background:**

Although immune system plays a key role in the pathogenesis of both endometriosis and ovarian cancer, its function is different. Therefore, we hypothesized, that selected immune parameters can serve as diagnostic markers of these two conditions. The aim of this study was to compare serum and peritoneal fluid concentrations of sHLA-G, IL-10 and TNF-alpha in women with selected ovarian pathologies: benign serous cysts, endometrioma and malignant tumors. Clinical significance of using them for diagnostic purposes in women with serous ovarian cysts, endometriosis, and ovarian cancer, which in the future may improve the early diagnosis of ovarian diseases.

**Case Presentation:**

The study included women treated surgically for benign serous ovarian cysts, ovarian endometrioma and serous ovarian adenocarcinomas. Peripheral blood and peritoneal fluid samples were obtained intraoperatively.

Patients with benign serous cysts, endometrioma and ovarian malignancies did not differ significantly in terms of their serum and peritoneal fluid concentrations of sHLA-G. Ovarian cancer patients presented with significantly higher median serum concentrations of IL-10 and TNF-alpha than other study subjects. Median concentrations of IL-10 and TNF-alpha in peritoneal fluid turned out to be the highest in ovarian cancer patients, followed by women with endometrioma and subjects with benign serous cysts. All these intergroup differences were statistically significant. Irrespective of the group, median concentrations of sHLA-G, IL-10 and TNF-alpha in peritoneal fluid were higher than serum levels of these markers.

**Conclusions:**

Elevated serum and peritoneal fluid concentrations of IL-10 and TNF-alpha distinguish ovarian malignancies and endometriomas from benign serous ovarian cysts. In contrast to endometriosis, ovarian malignancies are characterized by elevated peritoneal fluid concentrations of IL-10 and TNF-alpha, elevated serum concentrations of IL-10 and low serum levels of TNF-alpha. Serum and peritoneal fluid concentrations of sHLA-G have no diagnostic value in differentiating between ovarian malignancies and endometriomas.

## Background

Most ovarian cancers are diagnosed in patients older than 50 years. The majority of ovarian malignancies are of epithelial origin. Histopathological type is a determinant of tumor growth, its metastatic potential, response to treatment and prognosis [1–3]. It is estimated that up to 10–15% of ovarian cancers have a genetic background [4]. Due to specific metabolic (intensive cellular metabolism, rich vascularization, local inflammation and microinjuries associated with ovulation and retrograde menstruation) and topographic features of the ovaries (location within peritoneal cavity, enabling potential malignancies to grow uncompromised and to spread locally), ovarian tumors constitute an important clinical problem. Intraperitoneal dissemination is a typical feature of ovarian cancers. Metastatic lesions are usually well-vascularized and synthesize similar mediators to those produced by the primary tumor, i.e., vascular endothelial growth factor (VEGF), tumor necrosis factor alpha (TNF-alpha), interferon alpha (INF-alpha), interleukin 2 (IL-2) and metalloproteinases; all of them play a role in the development of malignant ascites [5].

Previous studies demonstrated that 10–15% of ovarian cancer patients present with concomitant endometriosis; both conditions develop under the same environmental conditions of minor pelvis [6, 7]. One of the key processes involved in the pathogenesis of endometriosis is activation of pro-inflammatory cytokines, which is responsible for most clinical manifestations of this condition [8].

Human leukocyte antigen G (HLA-G) is a major histocompatibility complex (MHC) antigen expressed on the cell surface. A total of seven isoforms of HLA-G have been identified thus far: membrane-bound HLA-G1, HLA-G2, HLA-G3 and HLA-G4, and soluble sHLA-G5, sHLA-G6 and sHLA-G7. While the biological effects of soluble and membrane-bound HLA-G are similar, the former are observed systemically. Expression of HLA-G on the surface of cancer cells is determined by a plethora of environmental factors, including hypoxia, stress, some hormones, cytokines and viruses [9]. HLA-G can activate T cells via a few various mechanisms. Interaction of HLA-G and CD4+ T cells with LILRB1 and LILRB2 receptors results in a decrease in the synthesis of T-helper 1 (Th1) cytokines, such as interferon gamma (INF-gamma), IL-2 and TNF-alpha, as well as in enhanced production of Th2 cytokines, among them interleukin 3, 4 and 10 (IL-3, IL-4 and IL-10). This results in inactivation of cytotoxic T cells and lesser production of anti-tumor antibodies [4, 10].

IL-10 is synthesized primarily by activated T cells, especially Th2and T regulatory cells (Tregs), inter alia in response to interaction of these cells with HLA-G/sHLA-G [11]. IL-10 is an important immunosuppressant and its elevated concentrations have been implicated in immune escape of some malignancies [12]. TNF-alpha interacts with cancer cells via its specific receptor; this results in activation of arachidonic acid cascade, enhanced generation of reactive oxygen species inside the cell and its death. However, this effect requires expression of specific receptors for TNF-alpha on the surface of cancer cells; otherwise this cytokine acts solely as an immunomodulatory agent [13].

The aim of this study was to compare serum and peritoneal fluid concentrations of sHLA-G, IL-10 and TNF-alpha in women with selected ovarian pathologies: benign serous cysts, endometrioma and malignant tumors. Moreover, we searched for correlations between serum and peritoneal fluid concentrations of these parameters. Clinical significance of using them for diagnostic purposes in women with serous ovarian cysts, endometriosis, and ovarian cancer, which in the future may improve the early diagnosis of ovarian diseases.

## Case Presentations

The study involved 135 women with suspected ovarian lesions, such as cysts, endometriosis of the small pelvis, and ovarian cancer, hospitalized in the obstetrics and gynecology ward in the Independent Public Specialist Healthcare Center “Zdroje” from 2009 to 2013. The final clinical diagnosis was based on the results of histopathological examination of the material taken during a surgery, which let us distinguish three groups of women. Group 1 (G1) consisted of 54 women with benign serous ovarian cysts; the mean age was 57 years. Group 2 (G2) consisted of 43 women with endometriosis of the peritoneum, the small pelvis, and/or the ovary; the mean age was 41 years. In the group with endometriosis (G2), stage I endometriosis was not observed (n-0), stage II endometriosis was diagnosed in 35 women (n-35), and stage III-IV endometriosis ― in 8 women (n-8). Group 3 (G3) comprised of 38 women with ovarian cancer; the mean age was 73 years. The patients in this group were either before (n-31) or during chemotherapy (n-7). In this group, ovarian carcinomas were divided into two types: Type I (*n* = 12), and Type II (*n* = 26). In the study groups, we excluded metastatic tumors from other organs, symptoms of infection, and thyroid diseases. What is more, the women did not receive steroid agents for 3 months preceding a surgery. According to the molecular criteria of Shih and Kurman [14], epithelial ovarian cancers can be divided into two types, namely type I and type II. Endometriosis-related cancers represent type I carcinogenesis. Cancers of this type develop slowly, are histologically well-differentiated, and thus show low mitotic potential. Borderline precursor lesions can often be observed in the course of their development. Type I tumors include well-differentiated serous cancers, mucinous and endometrioid ovarian cancers, clear-cell carcinomas, and transitional cell cancers, which make up to 25% of all ovarian malignancies. These cancers have a relatively stable genome, and the most common mutations are found in the *K-RAS*, *BRAF*, *PTEN*, *BCL-2*, and *ARID1A* genes. Type II cancers are tumors that spread rapidly, are primarily very advanced within the abdominal cavity and pelvis, and have an extremely aggressive clinical course without a previously noticeable precursor lesion. These carcinomas account for 75% of ovarian malignancies. They include poorly-differentiated serous cancers, non-differentiated cancers, and carcinosarcoma. Type II ovarian cancers are genetically unstable and are characterized by the presence of many mutations (the most frequent in the gene *p53*).

The revised American Fertility Society (rAFS) has divided endometrial involvement into 4 stages or degrees, depending on its size, infiltration, the presence of cysts and adhesions. Stage I denotes minimal endometriosis, II – mild endometriosis, III – moderate endometriosis, and IV – severe endometriosis [15]. The protocol of the study was approved by the Local Bioethics Committee at the Pomeranian Medical University in Szczecin, and written informed consent was sought from all the study subjects.

Blood was taken from the patients once before operation, and peritoneal fluid was taken once intraoperatively. All laboratory tests were performed at the Assisted Reproduction Laboratory, Reproductive Medicine and Gynecology Clinic in Police. Serum and peritoneal fluid concentrations of sHLA-G were determined by means of ELISA with commercially available kits from Bio Vendor Laboratory Medicine, Inc. (catalogue no. RD194070100R). Sensitivity threshold of this assay is 3 U/ml. Concentrations of IL-10 and TNF-alpha were measured by means of ELISA with specific monoclonal antibodies against these cytokines, using commercially available kits Quantikine and Quantikine HS from R&D Systems Europe, Ltd. The sensitivity thresholds of Quantikine and Quantikine HS assays are 3.9 pg/ml and 0.03–0.17 pg/ml (mean 0.9 pg/ml), respectively, for IL-10, and 0.5–5.5 pg/ml and 0.038–0.191 pg/ml (mean 1.6 pg/ml), respectively, for TNF-alpha.

All statistical calculations were carried out with STATA 11 software (license no. 30110532736). Normal distributions of continuous variables were verified with Kolmogorov-Smirnov test. Statistical characteristics of quantitative variables are presented as medians, lower and upper quartiles. Depending on the distribution type and the number of analyzed groups, statistical significance of intergroup differences was verified with Student *t*-test for independent variables, Mann-Whitney *U*-test, analysis of variance (ANOVA) or Kruskal-Wallis test. The significance of intragroup differences in serum and peritoneal levels of analyzed parameters was verified with either Student *t*-test for dependent variables or Wilcoxon test. Power and direction of relationships between pairs of quantitative variables were determined on the basis of Pearson’s coefficients of linear correlation or Spearman’s coefficients of rank correlation. All tests were considered significant at *p* ≤ 0.05.

**Fig. 1:**
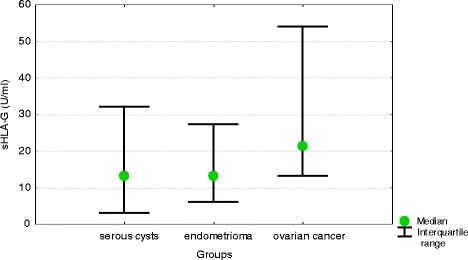
Differences between peritoneal fluid and serum concentrations of sHLA-G in women with benign serous ovarian cysts, ovarian endometrioma and ovarian cancer (medians and interquartile ranges)

Patients with benign serous cysts, endometrioma and ovarian malignancies did not differ significantly in terms of their serum and peritoneal fluid concentrations of sHLA-G (Table 1). Median difference between peritoneal fluid and serum concentrations of sHLA-G in ovarian cancer patients (21.2 U/ml) turned out to be significantly higher than in women with benign serous cysts (13.04 U/ml) and endometrioma (13.00 U/ml) (Fig. 1).

**Table 1:** Serum and peritoneal fluid concentrations of sHLA-G, IL-10 and TNF-alpha in women with benign serous ovarian cysts, ovarian endometrioma and ovarian cancer (medians and interquartile ranges)

Parameter	Serous cysts (*n* = 54)	Endometrioma (*n* = 43)	Ovarian cancer (*n* = 38)	*p*
Serum sHLA-G (U/ml)	8.90 (5.35–21.75)	7.70 (5.15–17.00)	10.00 (5.47–14.40)	0.944
Peritoneal fluid sHLA-G (U/ml)	29.75 (15.63–51.70)	20.60 (15.80–37.54)	36.15 (20.68–62.08)	0.150
Serum IL-10 (pg/ml)	3.75^*^ (3.20–11.20)	4.60^*^ (4.20–6.65)	11.30 (4.60–21.00)	0.002
Peritoneal fluid IL-10 (pg/ml)	18.15^#,§^ (4.60–41.70)	53.00^*^ (14.70–93.20)	128.80 (36.71–252.38)	<0.001
Serum TNF-alpha (pg/ml)	1.20^#^ (1.00–1.73)	1.00^#^ (0.73–1.68)	2.70 (1.90–4.00)	<0.001
Peritoneal fluid TNF-alpha (pg/ml)	1.80^#,§ ^(1.00–3.80)	3.10^‡^ (1.70–17.50)	13.21 (8.35–25.35)	<0.001

Significantly lower than in ovarian cancer patients: ^*^ *p* < 0.005, ^‡^ *p* < 0.001, ^#^ *p* < 0.0001; significantly lower than in endometrioma patients: ^§^ *p* < 0.005

**Fig. 2:**
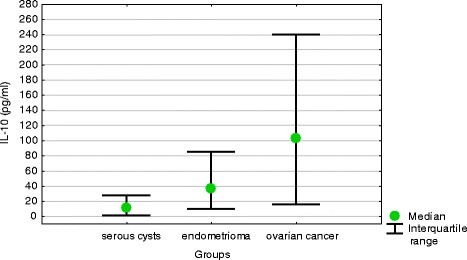
Differences between peritoneal fluid and serum concentrations of IL-10 in women with benign serous ovarian cysts, ovarian endometrioma and ovarian cancer (medians and interquartile ranges)

Ovarian cancer patients presented with significantly higher median serum concentrations of IL-10 than other study subjects. Also median concentrations of IL-10 in peritoneal fluid turned out to be the highest in ovarian cancer patients, followed by women with endometrioma and subjects with benign serous cysts. All these intergroup differences were statistically significant (Table 1). Irrespective of the group, median concentrations of IL-10 in peritoneal fluid were higher than serum levels of this cytokine. Median difference between peritoneal fluid and serum concentrations of IL-10 was the highest in ovarian cancer patients, followed by women with endometrioma and those with serous ovarian cysts. All these intergroup differences were statistically significant (Fig. 2).

**Fig. 3:**
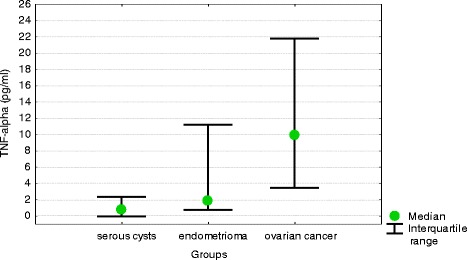
Differences between peritoneal fluid and serum concentrations of TNF-alpha in women with benign serous ovarian cysts, ovarian endometrioma and ovarian cancer (medians and interquartile ranges)

Median serum concentrations of TNF-alpha in ovarian cancer patients were significantly higher than in other study subjects. Also median concentrations of TNF-alpha in peritoneal fluid were the highest in ovarian cancer patients, followed by women with endometrioma and those with benign serous cysts. All these intergroup differences turned out to be significant on statistical analysis (Table 1). Also median difference between peritoneal fluid and serum concentrations of TNF-alpha was the highest in ovarian cancer patients, followed by women with endometrioma and serous ovarian cysts. All these intergroup differences were statistically significant (Fig. 3).

**Fig. 4:**
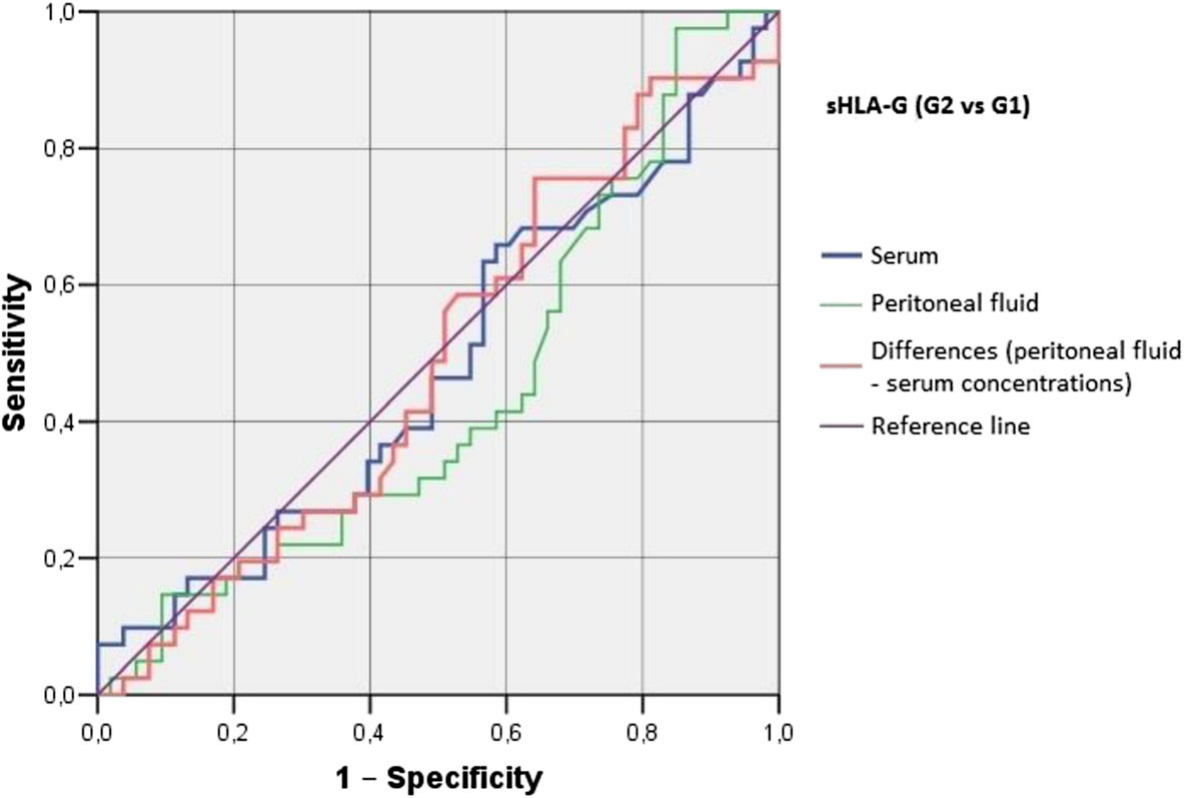
The ROC graph showing serum and peritoneal sHLA-G concentrations, and the difference in peritoneal and serum concentrations between G2 and G1 groups

**Fig. 5:**
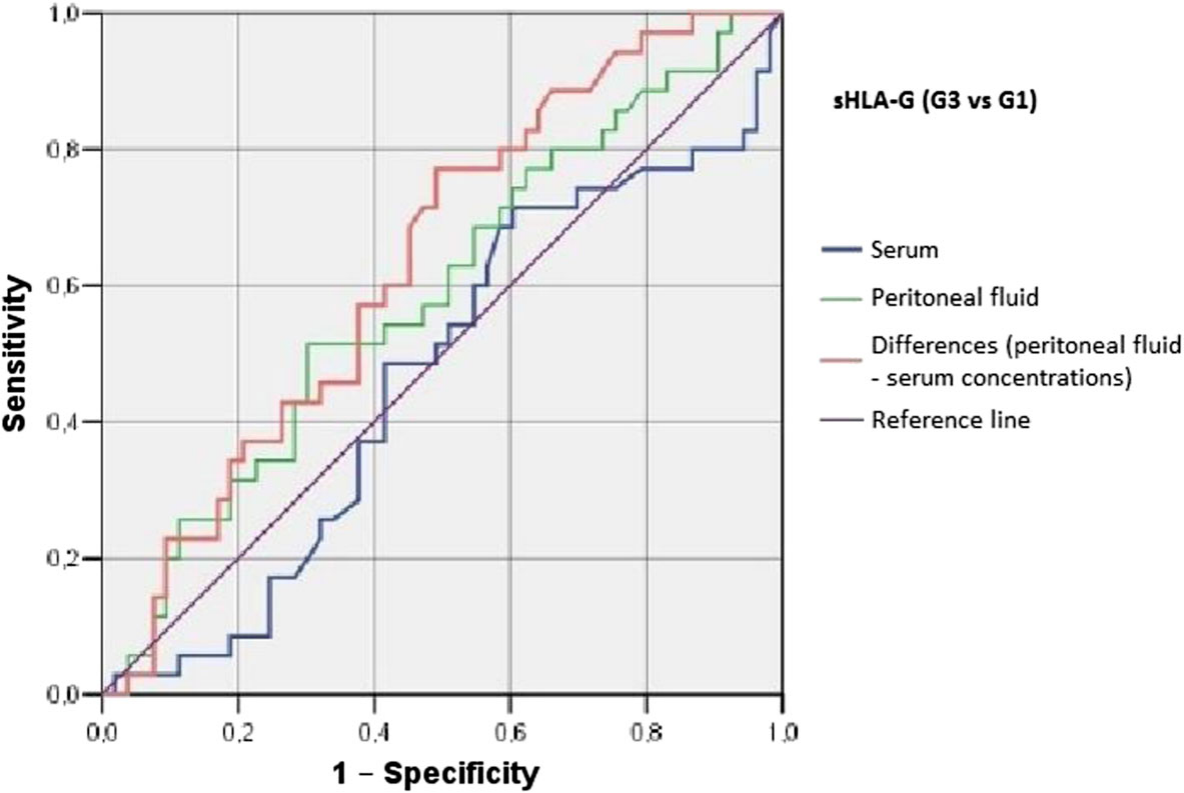
The ROC graph showing serum and peritoneal sHLA-G concentrations, and the difference in peritoneal and serum concentrations between G3 and G1 groups

**Fig. 6:**
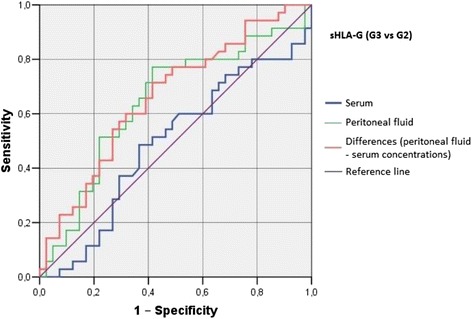
The ROC graph showing serum and peritoneal sHLA-G concentrations, and the difference in peritoneal and serum concentrations between G3 and G2 groups

**Fig. 7:**
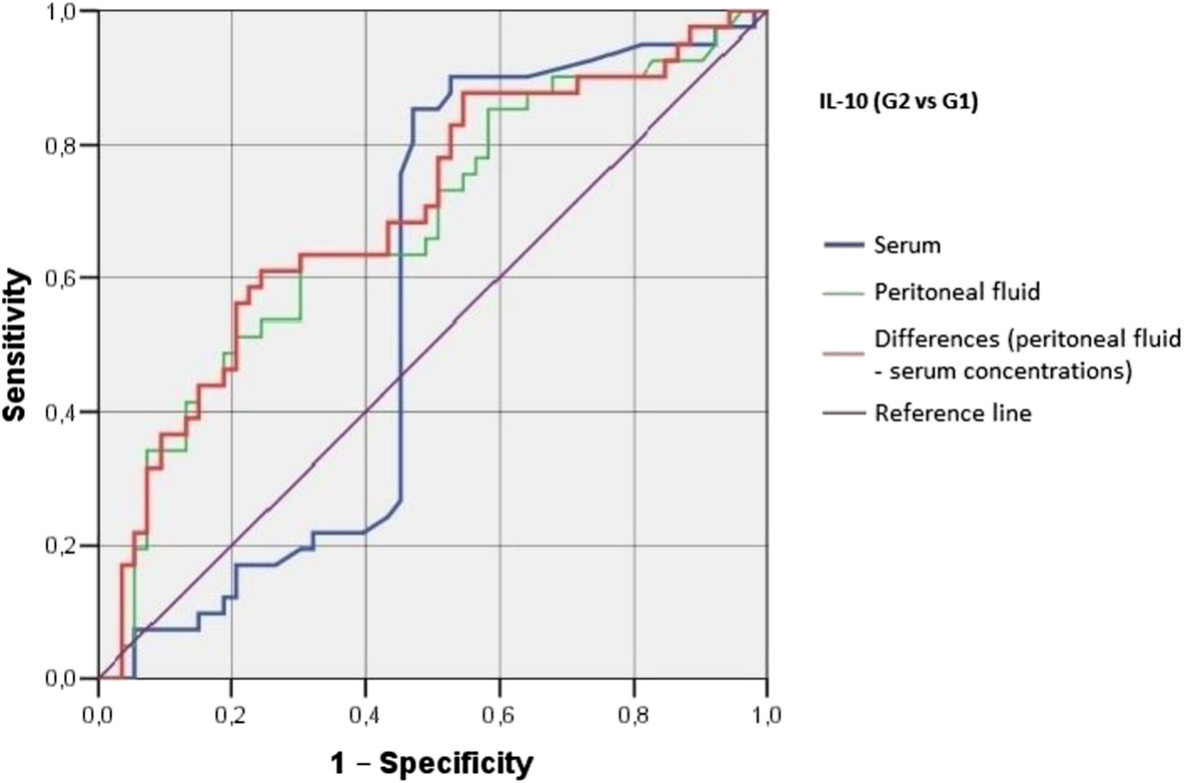
The ROC graph showing serum and peritoneal IL-10 concentrations, and the difference in peritoneal and serum concentrations between G2 and G1 groups

**Fig. 8:**
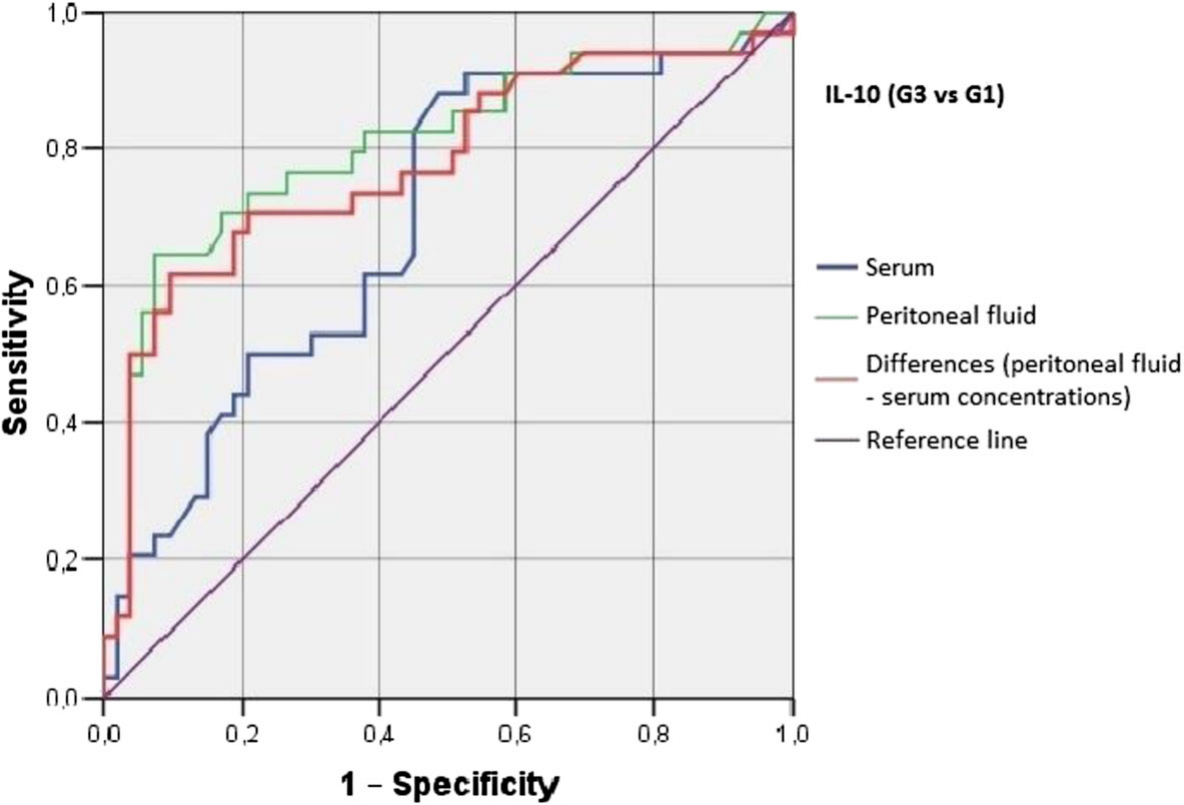
The ROC graph showing serum and peritoneal IL-10 concentrations, and the difference in peritoneal and serum concentrations between G3 and G1 groups

**Fig. 9:**
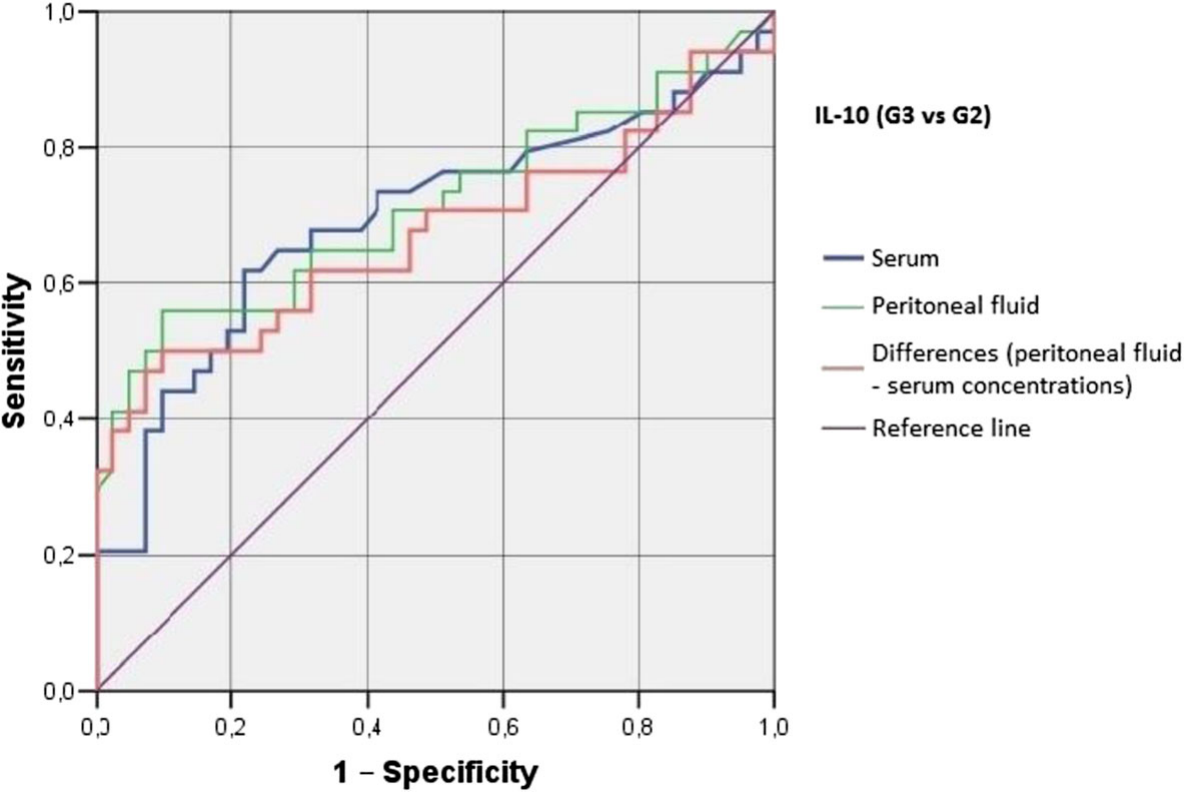
The ROC graph showing serum and peritoneal IL-10 concentrations, and the difference in peritoneal and serum concentrations between G3 and G2 groups

Irrespective of the group, we did not find statistically significant correlations between serum/peritoneal fluid concentrations of sHLA-G and concentrations of IL-10 and TNF-alpha in these materials. Serum concentration of IL-10 in women with benign serous cysts correlated positively with peritoneal fluid concentration of this cytokine and serum concentration of TNF-alpha; furthermore, a positive correlation between peritoneal fluid levels of IL-10 and TNF-alpha was found in this group. The only statistically significant correlation observed in endometrioma patients was a positive association between concentrations of IL-10 and TNF-alpha in peritoneal fluid. Serum concentration of IL-10 in ovarian cancer patients correlated positively with peritoneal fluid concentration of this cytokine and serum level of TNF-alpha; moreover, women with ovarian malignancies showed a positive correlation between serum TNF-alpha an IL-10 in peritoneal fluid (Table 2). Analysis of the diagnostic sensitivity of the tested laboratory parameters in serum and peritoneal fluid. The parameters that had the highest diagnostic value, expressed as AUC > 0.6, allowing for differentiating between G2 and G3 groups, were peritoneal sHLA-G concentrations, and the difference between sHLA-G concentrations in serum and peritoneal fluid; sensitivity > 70%, and the level of significance *p* < 0.05. The ROC graphs in Figs. 4, 5 and 6 indicate to the usefulness of peritoneal sHLA-G concentrations as a parameter differentiating between G2 and G3 groups. The cut-off peritoneal concentrations of sHLA-G ≥ 22.1 U/ml, and the difference between peritoneal and serum sHLA-G concentrations ≥ 14.25 U/ml potentially indicate to ovarian cancer, while lower values suggest endometriosis. The highest differentiating value for G1 and G3 groups was attributed to peritoneal IL-10 concentrations, with a cut-off value of 34.5 pg/ml, and to the difference between peritoneal and serum IL-10 concentrations, with a cut-off value of 27.6 pg/ml. The most sensitive parameter (89%, *p* < 0.002) differentiating between G1 and G3 groups was serum IL-10 concentration, with a cut-off value of 3.8 pg/ml (Figs. 7, 8 and 9).

**Table 2:** Correlations between serum and peritoneal fluid concentrations of sHLA-G, IL-10 and TNF-alpha in women with benign serous ovarian cysts, ovarian endometrioma and ovarian cancer (Pearson’s coefficients of linear correlation or Spearman’s coefficients of ran correlation)

Variable	Peritoneal sHLA-G	Serum IL-10	Peritoneal IL-10	Serum TNF-alpha	Peritoneal TNF-alpha
Serous cysts (*n* = 54)					
Serum sHLA-G	0.07 (*p* = 0.635)	−0.19 (*p* = 0.178)	−0.07 (*p* = 0.637)	0.13 (*p* = 0.371)	−0.23 (*p* = 0.116)
Peritoneal sHLA-G		0.13 (*p* = 0.362)	0.16 (*p* = 0.253)	−0.10 (*p* = 0.483)	0.06 (*p* = 0.662)
Serum IL-10			0.42 (*p* = 0.002)	0.35 (*p* = 0.013)	0.14 (*p* = 0.322)
Peritoneal IL-10				0.09 (*p* = 0.527)	0.42 (*p* = 0.002)
Serum TNF-alpha					0.22 (*p* = 0.133)
Endometrioma (*n* = 43)					
Serum sHLA-G	0.15 (*p* = 0.348)	−0.06 (*p* = 0.715)	−0.06 (*p* = 0.724)	−0.04 (*p* = 0.800)	−0.06 (*p* = 0.714)
Peritoneal sHLA-G		0.05 (*p* = 0.754)	0.10 (*p* = 0.517)	0.26 (*p* = 0.110)	−0.02 (*p* = 0.875)
Serum IL-10			0.15 (*p* = 0.350)	0.11 (*p* = 0.495)	−0.16 (*p* = 0.318)
Peritoneal IL-10				0.09 (*p* = 0.581)	0.50 (*p* = 0.001)
Serum TNF-alpha					0.25 (*p* = 0.123)
Ovarian cancer (*n* = 38)					
Serum sHLA-G	0.15 (*p* = 0.389)	0.04 (*p* = 0.806)	−0.19 (*p* = 0.270)	0.22 (*p* = 0.201)	−0.03 (*p* = 0.860)
Peritoneal sHLA-G		−0.17 (*p* = 0.328)	−0.11 (*p* = 0.500)	−0.12 (*p* = 0.511)	0.21 (*p* = 0.205)
Serum IL-10			0.50 (*p* = 0.003)	0.50 (*p* = 0.003)	−0.12 (*p* = 0.501)
Peritoneal IL-10				0.40 (*p* = 0.021)	0.27 (*p* = 0.106)
Serum TNF-alpha					0.27 (*p* = 0.126)

**Fig. 10:**
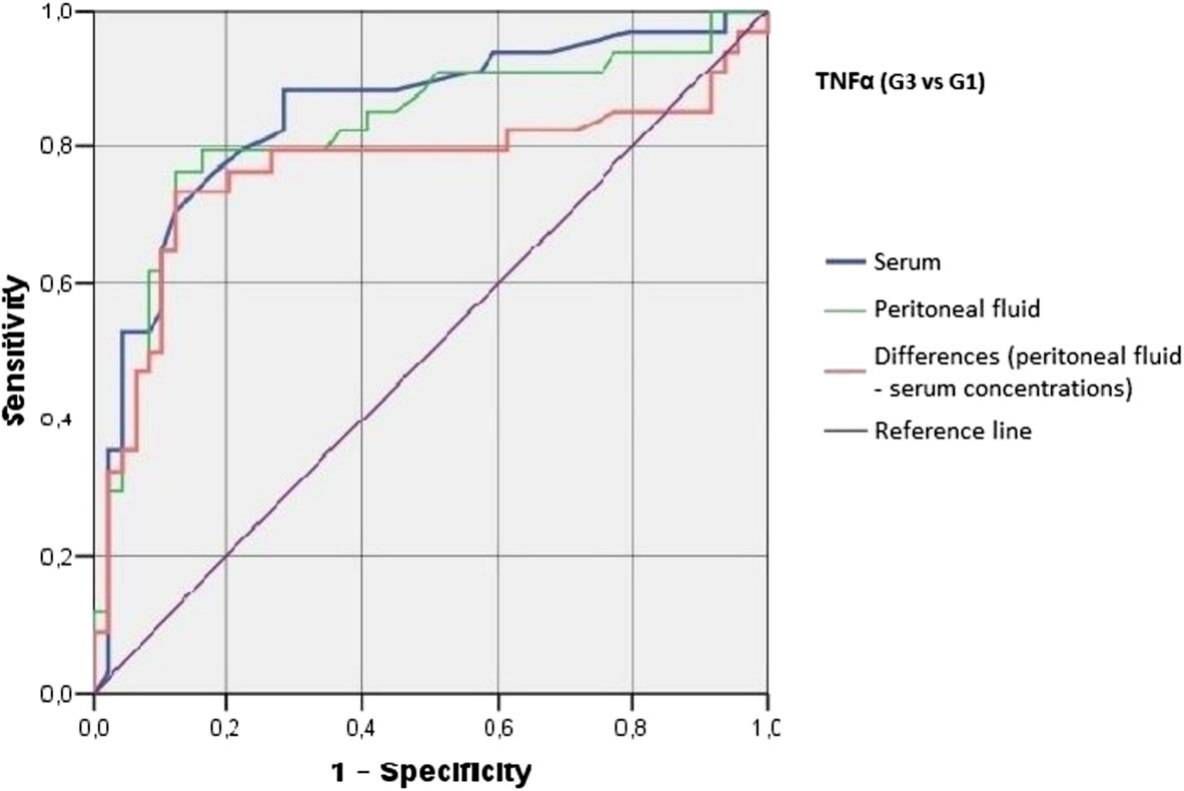
The ROC graph showing serum and peritoneal TNF-alpha concentrations, and the difference in peritoneal and serum concentrations between G2 and G1 groups

**Fig. 11:**
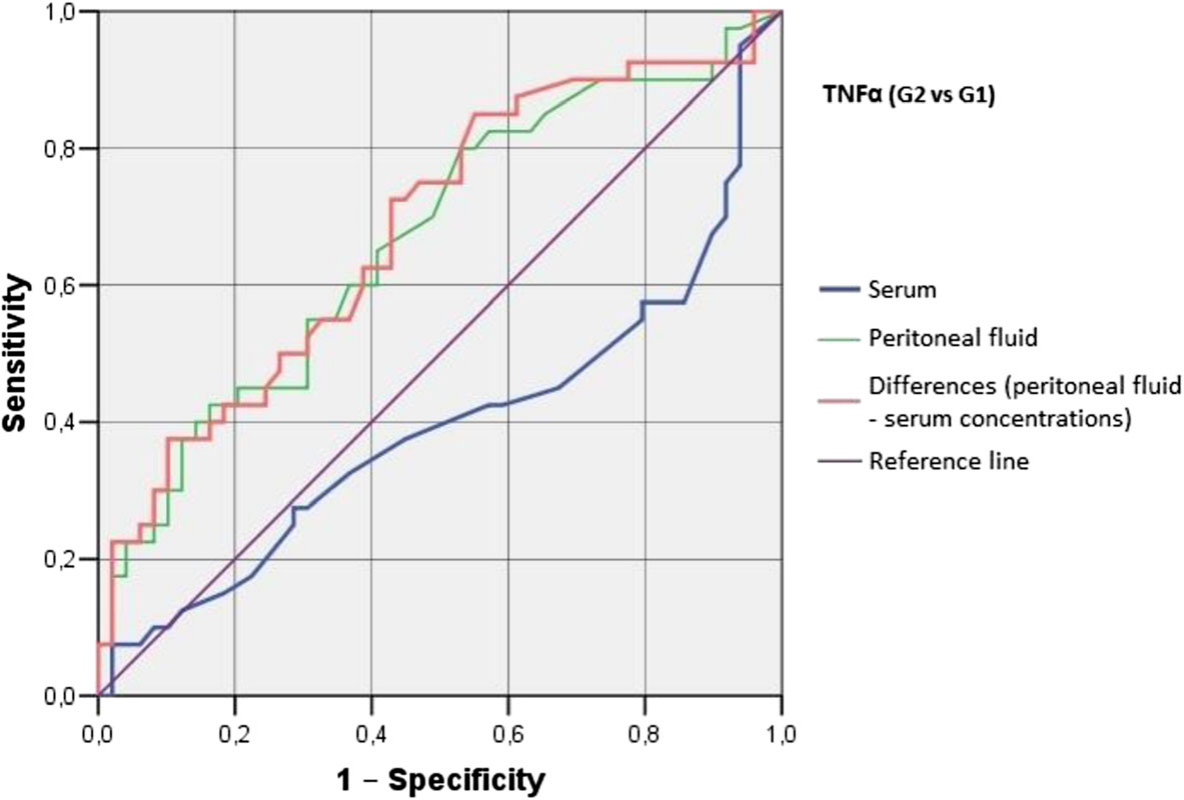
The ROC graph showing serum and peritoneal TNF-alpha concentrations, and the difference in peritoneal and serum concentrations between G3 and G1 groups

**Fig. 12:**
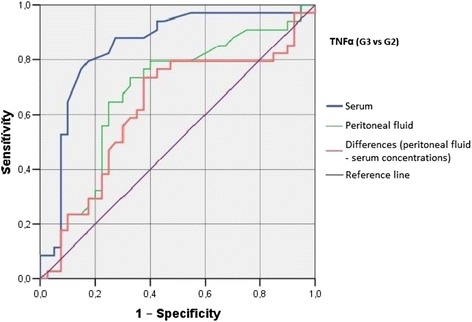
The ROC graph showing serum and peritoneal TNF-alpha concentrations, and the difference in peritoneal and serum concentrations between G3 and G2 groups

G1 and G3 groups, and G2 and G3 groups were best differentiated by serum and peritoneal TNF-alpha concentrations, with cut-off values of 1.55 pg/ml (G1 vs. G3) and 1.75 pg/ml (G2 vs. G3). The concentration of TNF-alpha in serum, with a cut-off value of 1.55 pg/ml, was the most sensitive differentiating parameter (89%, *p* < 0.0001) between G1 and G3 groups. G1 and G3 groups can also be differentiated by measuring serum and peritoneal TNF-alpha concentrations, with a cut-off value of 6.75 pg/ml (sensitivity of 74%, specificity of 88%) (Figs. 10, 11 and 12). Only peritoneal TNF-alpha concentrations, and the difference between peritoneal and serum TNF-alpha concentrations significantly differentiate between G1 and G2 groups.

## Discussion

Ovarian malignancies represent a challenge in gynecologic oncology. This results primarily from problems with identification of high risk groups, asymptomatic character of the disease at its early stages and lack of sufficiently specific and sensitive tests suitable for population based screening. In line with current standards, aside from clinical examination and imaging studies, evaluation of patients with suspected ovarian masses should also include determination of cancer markers [13].

Concentrations of potential cancer markers (sHLA-G, IL-10 and TNF-alpha) in body fluids (serum and peritoneal fluid) are not stable, which may be associated with both their sources and natural history of the disease. Serum concentrations of sHLA-G, IL-10 and TNF-alpha reflect systemic immune activity associated with underlying condition, whereas their levels in peritoneal fluid are associated rather with local processes.

A number of previous studies documented a link between HLA-G/sHLA-G and carcinogenesis [16–18]. Rouas-Freiss et al. [19] found expression of HLA-G on both cancer cells and tumor-infiltrating immune cells. They showed that it is the tumor itself which stimulates the expression of HLA-G/sHLA-G on both cancer cells and infiltrating immune cells, especially CD68+ macrophages and CD8+ T cells. However, they did not observe expression of these antigens in normal tissues around the tumor. Cancer patients show enhanced expression of HLA-G/sHLA-G both locally and in the blood serum. Consequently, the increase in sHLA-G concentrations in peritoneal fluid and serum of our ovarian cancer patients is consistent with previously published data [20].

Soluble HLA-G was first found in the trophoblast whereby it exerted an immunosuppressive effect, protecting fetus, recognized as a foreign antigen, against maternal immune response. Tumors and trophoblast share some common features, such as high proliferation rate, invasiveness, synthesis of growth factors and their receptors, hormones and proto-oncogenes. Furthermore, previous studies found some similarities in the immunosuppressive effects of sHLA-G expressed by pregnant women and cancer patients. According to Mach [21], expression of this antigen in cancer patients is associated with progression of the malignancy and worse prognosis. Basta et al. [22] analyzed the role of HLA-G in progression of recurrent ovarian cancer. They found expression of this antigen both on cancer cells and on macrophages infiltrating the tumor and its microenvironment. The study conducted by these authors included two groups of women with recurrent ovarian cancer, with complete remission after primary treatment and without. The latter group presented with higher concentrations of HLA-G within the tumor, which implies that expression of this antigen may be associated with cancer stage.

Our findings are also consistent with the results presented by Matalliotakis et al. [23], according to whom women with active endometriosis present with lower serum levels of sHLA-G. The same study showed that serum level of sHLA-G normalizes after treatment and then decreases again whenever the symptoms of endometriosis recur. Our results are also in line with those reported by Eidukaite and Tamosiunas [24], who found that concentrations of sHLA-G in peritoneal fluid from women with endometriosis and without are similar. Barrier et al. [25] demonstrated that whereas HLA-G is expressed by endometroid cells, it is absent in normal endometrial cells. However, Wang et al. [26] found the expression of HLA-G in both normal and ectopic endometrium, and Hornung et al. [27] did not detect this antigen in either normal endometrium or endometrial foci. All these discrepancies may reflect differences in the analyzed material and examined parameters (HLA-G vs. sHLA-G).

In our study, women with serous ovarian cysts showed a considerable variability in both serum and peritoneal fluid concentrations of sHLA-G, and no significant intergroup differences were found with regards to these parameters. To the best of our knowledge, there is no published evidence documenting a link between sHLA-G concentration and serous ovarian cysts. However, it should be remembered that concentration of sHLA-G in patients with various conditions may change across their menstrual cycle; this might affect our findings and contribute to a relatively low discriminatory power of this parameter. In line with the surgical protocol, material from women with endometriosis was collected during the 1^st^ phase of menstrual cycle. In turn, the time of sampling in patients with serous ovarian cysts was highly variable and some of these women were amenorrheic. Mach et al. [28] analyzed phase-specific changes in serum concentrations of sHLA-G in patients with endometriosis and ovarian carcinomas. They found that irrespective of the group, serum concentrations of sHLA-G were always higher during the 1^st^ phase of the cycle. However, while the levels of sHLA-G in patients with ovarian cancer and deep endometriosis remained elevated during further stages of the cycle as well, a significant decrease in this parameter was observed during the secretory phase in women with ovarian endometrioma. This evidence points to the 1^st^ phase of menstrual cycle as an optimal timing to determine serum concentration of sHLA-G.

Clinical importance of HLA-G/sHLA-G is a subject of an extensive research, as shown by a plethora of recently published papers documenting biological effects of these antigens. Sheu and Shih [17] described the mechanisms via which sHLA-G interacts with immune cells. Dimerics HLA-G impairs the ability of dendritic cells to present antigen, activates suppressor lymphocytes and inhibits synthesis of interleukin 12 (IL-12). In turn, sHLA-G monomer is responsible for functional inhibition of B cells due to induction of their apoptosis and promotion of Th2 cytokine (IL-10, IL-3 and IL-4) production. Due to their immunosuppressive character, these cytokines impair anti-inflammatory and anti-tumor response. The same authors [18] found expression of HLA-G on ovarian cancer cells and documented its role in immune escape of this malignancy.

We did not find significant differences in serum concentrations of sHLA-G in patients with benign serous ovarian cysts, endometrioma and ovarian tumors. Based on available evidence we expected that patients with endometrioma and ovarian malignancies will present with lower and higher levels of sHLA-G, respectively. However, this hypothesis was not confirmed, probably due to a substantial variability of individuals results in both groups. Also the difference in serum concentrations of sHLA-G in patients with endometriomas and serous ovarian cysts was not statistically significant. A number of factors may explain the lack of significant intergroup differences. First, as already mentioned, HLA-G is present in both membrane-bound and soluble form. Although both of them exert similar biological effects, it should be remembered that our study was limited solely to sHLA-G. Another potential explanation for the lack of higher concentrations of sHLA-G in ovarian cancer patients may be the fact that this antigen is expressed only by some ovarian cancer lines. Singer et al. [20] examined sHLA-G as a potential marker of ascites; they showed that although the sensitivity of this parameter in the differential diagnosis of malignant and benign ascites is similar as that of cytological examination, contrary to the former it cannot be used to establish an ultimate diagnosis. Consequently, determination of sHLA-G seems to be primarily applicableto research on the pathomechanisms of ovarian cancer and endometriosis.

Presence of tumor is associated with local inflammation resulting from infiltration with immune cells, autocrine and paracrine release of cytokines. Moradi et al. [29] analyzed concentrations of TNF-alpha, interleukin 1 and 6(IL-1and IL-6) in serum and peritoneal fluid from healthy women and ovarian cancer patients, and demonstrated that presence of malignancy was associated with a significant increase in all these parameters. The increase in TNF-alpha level results from activation of immune response to ovarian tumor. The synthesis of this cytokine depends on tumor-specific cells, primarily tumor-associated macrophages. Kulbe et al. [30] found elevated concentrations of pro-inflammatory cytokines, such as TNF-alpha, C-X-C motif chemokine ligand 12 (CXCL12) and IL-6 in many cancer cell lines, and documented their role in angiogenesis and infiltration. Our findings are consistent with observations of these authors, pointing to a link between TNF-alpha and carcinogenesis. Complex role of TNF-alpha in tumor pathogenesis was a subject of many studies; their authors tried to explain why this pro-inflammatory cytokine either contributes to cancer control or promotes its growth and spread.

Endometriosis is characterized by chronic inflammation resulting from synthesis of many pro-inflammatory and immunosuppressive cytokines. Interactions between various cytokines, including TNF-alpha, are responsible for aggravation of inflammatory processes, resultant hyperemia, pain, tissue injury, formation of adhesions and dysfunction of various organs and systems. One of the consequences is impaired fertility. Koninckxet al. [31] analyzed the role of TNF-alpha in pelvic inflammation and pain associated with endometriosis, and verified if these ailments may be attenuated with anti-TNF monoclonal antibodies.

Our observation that women with endometriomas present with higher peritoneal fluid concentrations of TNF-alpha is consistent with the results of previous studies conducted by Eisermann et al. [32], Braun et al. [33] and Rana et al. [34]. Also according to Podgaec et al. [35], women with endometriosis present with elevated peritoneal fluid concentrations of TNF-alpha and other Th1 cytokines, as well as which higher levels of IL-10 and other Th2 cytokines. In turn, Xavier et al. [36] demonstrated that irrespective of the cycle phase, endometriosis is associated with elevated serum concentrations of TNF-alpha. According to Zhang et al. [37], the increase in TNF-alpha level promotes adhesion of endometrial cells to peritoneum both in vitro and in vivoand therefore, may play a role in the pathogenesis of endometriosis. Similar to our study, also other authors reported elevated serum concentrations of TNF-alpha in women with endometriosis and ovarian cancer [37–39]. Bedaiwy et al. [38] compared diagnostic value of CA125 and other serum, peritoneal fluid, tissue and genetic markers of endometriosis; the only parameters suitable for differential diagnosis of this condition were concentration of TNF-alpha in peritoneal fluid and serum level of IL-6. In turn, Galo et al. [40] demonstrated that women with endometriosis present with elevated serum levels of TNF-alpha and in contrary to our study, identified this parameter as an accurate diagnostic marker of this condition.

According to Zhou et al. [41], peritoneal fluid concentration of IL-10 is significantly higher than serum concentration of this cytokine. The same authors demonstrated that ovarian cancer patients present with significantly higher values of this parameter than women with benign lesions, which is also consistent with our findings. Furthermore, they observed that concentration of IL-10 in ovarian cancer cell lysate was markedly higher than its serum level, which points to tumor microenvironment as a principal source of this cytokine. According to Berger et al. [42], IL-10 can be synthesized by various ovarian cancer cell lines. Berek et al. [43] documented lower cytotoxicity of lymphocytes isolated from serum and peritoneal fluid of ovarian cancer patients. One potential explanation for this phenomenon was provided by Rouas-Freiss et al. [19]; similar to our study, these authors demonstrated that ovarian cancer patients present with elevated serum levels of IL-10 and other immunomodulatory cytokines, such as leukemia inhibitory factor (LIF), granulocyte macrophage colony stimulating factor (GM-CSF) and IFN-gamma.

Similar to our study, Mustea et al. [44] demonstrated that ovarian cancer patients, especially those with serous tumors, present with elevated peritoneal fluid concentrations of IL-10, markedly higher than serum levels of this cytokine. Higher concentrations of IL-10 were associated with more advanced clinical stages of ovarian cancer, which was also reported by Cho and Shih [2]. Elevated concentrations of IL-10 in ascites fluid from ovarian cancer patients were also reported by Yigit et al. [45] and Matte et al. [46]. These findings imply that due to its immunosuppressive effects, IL-10 likely interferes with both systemic and local anti-tumor response and promotes progression of ovarian malignancies.

Mutual interactions between cytokines, their influence on the cells of the immune system and the expression of surface antigens, as well as multifactor regulation of their secretion cause that sHLA-G, IL-10 and TNF-alpha are functionally related and their concentrations can correlate with each other. Cho [2] as well as Sheu and Shih [18] found that high expression of HLA-G/sHLA-G and a high level of IL-10 in the micro-environment of ovarian cancer contribute to an aggressive course of the disease. They explained the mechanisms of mutual interactions between various forms of HLA-G and immune system cells. Bukur et al. [47] described the molecular basis of HLA-G expression in various neoplasms, and immunosuppressive interactions between sHLA-G and IL-10 in kidney cancer.

Yoon et al. [48] analyzed the expression of HLA-G and its impact on the IL-10 concentration in cervical cancer, and reported on a relationship between the produced IL-10 and sHLA-G expression.

A similar relationship may also exist between TNF-alpha and IL-10, as they represent two opposite but complementary types of immunological response. After primary activation of proinflammatory processes, characterized by the presence of TNF-alpha, the inflammatory response is suppressed through the activation of immunosuppressive mechanisms and the release of IL-10. In chronic inflammatory states, these cytokines exist next to each other, competing in the micro-environment in a peculiar way.

Cassatella et al. [49] studied inhibiting effects of IL-10 on the production of TNFα through neutrophils stimulated with lipopolysaccharide, and noticed that the production of TNF-alpha was inhibited proportionally to an increase in IL-10 concentration, which suggests that there is a negative correlation between TNF-alpha and IL-10. Kanai et al. [50] demonstrated that soluble HLA-G stimulates the release of TNF-alpha and INFγ by peripheral blood mononuclear cells, and reduces IL-3 secretion, suggesting that sHLA-G and TNF-alpha are correlated, which, however, was not confirmed by our findings. Summing up, our study shows that in most cases, concentrations of the tested indicators are not related to each other. Hence a diagnostic justification for measuring each of them.

Kleinberg et al. [51] studied the relationship of HLA-G to breast cancer and pleural mesothelioma. When analyzing the tumor and effusion fluid in its area, they found that the prognostic value of sHLA-G expression can differ depending on the type of neoplasm.

Similarly, Ye et al. [52] investigated HLA-G expression and assessed its prognostic value in patients with colorectal cancer, finding it to be an independent prognostic factor in these patients.

Sebti et al. [53] and Gros et al. [54], who gauged immunological significance of sHLA-G in patients with chronic lymphocytic leukemia, obtained comparable results.

In our study, only slight differences in serum sHLA-G levels were observed between the groups of women. It is true that sHLA-G levels were low in the group with endometriosis and high in the group with ovarian cancer. Nevertheless, discrepancies in sHLA-G levels within each of these groups caused that they did not differentiate ovarian cancer from non-cancerous lesions. The difference in sHLA-G levels between the group with endometriosis and the group with serous cysts was not statistically significant. In the organism, HLA-G can occur in membrane-bound and soluble forms, both of them having a similar biological effect. In our study, only the soluble form of HLA-G was taken into account. sHLA-G is only present in some ovarian cancer cell lines, therefore in many cases of this disease its concentration is low.

Singer et al. [20] made an attempt at using sHLA-G as a tumor marker to diagnose ascites, and achieved sensitivity comparable with cervical cytology. However, unlike cervical cytology, the use of sHLA-G gave no final, unambiguous diagnosis.

It seems that measuring sHLA-G levels is mainly of cognitive value in the analysis of the pathomechanisms involved in the development of ovarian cancer and endometriosis.

Bedaiwy et al. [38] compared the use of Ca125 in detecting endometriosis with various serum, peritoneal fluid, tissue, and genetic markers, and concluded that only TNF-alpha in peritoneal fluid and IL-6 in serum were of significant diagnostic value.

Likewise, Galo et al. [40] observed that women with endometriosis had increased serum TNF-alpha levels. In their prospective clinical research, these authors found that TNF-alpha in serum was a good marker for endometriosis, which was not supported by our findings, showing no significant differences between the groups.

## Conclusions

Elevated serum and peritoneal fluid concentrations of IL-10 and TNF-alpha distinguish ovarian malignancies and endometriomas from benign serous ovarian cysts. In contrast to endometriosis, ovarian malignancies are characterized by elevated peritoneal fluid concentrations of IL-10 and TNF-alpha, elevated serum concentrations of IL-10 and low serum levels of TNF-alpha. Serum and peritoneal fluid concentrations of sHLA-G have no diagnostic value in differentiating between ovarian malignancies and endometriomas.

## Abbreviations

ANOVA: Analysis of variance
CXCL12: C-X-C motif chemokine ligand 12
GM-CSF: Granulocyte macrophage colony stimulating factor (GM-CSF)
HLA-G: Human leukocyte antigen
IFN-alpha: Interferon alpha
IFN-gamma: Interferon gamma
IL-10: Interleukin 10
IL-12: Interleukin 12
IL-2: Interleukin 2
IL-3: Interleukin 3
IL-4: Interleukin 4
LIF: Leukemia inhibitory factor
MHC: Major histocompatibility complex
sHLA-G: Soluble human leukocyte antigen
Th cells: T-helper cells
TNF-alpha: Tumor necrosis factor alpha
Tregs: T-regulatory cells
VEGF: Vascular endothelial growth factor
